# A Comparative Analysis of Shoes Designed for Subjects with Obesity Using a Single Inertial Sensor: Preliminary Results

**DOI:** 10.3390/s22030782

**Published:** 2022-01-20

**Authors:** Veronica Cimolin, Michele Gobbi, Camillo Buratto, Samuele Ferraro, Andrea Fumagalli, Manuela Galli, Paolo Capodaglio

**Affiliations:** 1 Department of Electronics, Information and Bioengineering, Politecnico di Milan, Piazza Leonardo da Vinci 32, 20133 Milan, Italy; veronica.cimolin@polimi.it (V.C.); manuela.galli@polimi.it (M.G.); 2 Orthopaedic Rehabilitation Unit and Research Lab for Biomechanics, Rehabilitation and Ergonomics, Ospedale San Giuseppe, Istituto Auxologico Italiano, IRCCS, via Cadorna 90, 28824 Piancavallo di Oggebbio, Italy; m.gobbi@auxologico.it (M.G.); a.fumagalli@auxologico.it (A.F.); 3 Podartis SRL, via Erizzo 123/c, 31035 Piancavallo, Italy; camillo.buratto@podartis.it (C.B.); samuele.ferraro@podartis.it (S.F.); 4 Department Surgical Sciences, Physical and Rehabilitation Medicine, University of Torino, 10126 Torino, Italy

**Keywords:** obesity, walking, 6-min walking test, wearable system, inertial sensor, rehabilitation

## Abstract

Walking remains a highly recommended form of exercise for the management of obesity. Thus, comfortable and adequate shoes represent, together with the prescription of a safe adapted physical activity, an important means to achieve the recommended physical activity target volume. However, the literature on shoes specific for obese individuals is inadequate. The aim of the present study was to compare the performance of shoes specifically designed for subjects with obesity with everyday sneakers during instrumented 6-min walking test and outdoor 30-min ambulation in a group of subjects with obesity using a single wearable device. Twenty-three obese individuals (mean age 58.96 years) were recruited and classified into two groups: deconditioned (n = 13) and non-deconditioned patients (n = 10). Each participant was evaluated with his/her daily sneakers and the day after with shoes specifically designed for people with obesity by means of a questionnaire related to the comfort related to each model of shoes and instrumentally during the i6MWT and an outdoor walking test. The results showed that the specifically designed shoes displayed the higher score as for comfort, in particular in the deconditioned group. During the i6MWT, the distance walked, and step length significantly increased in the deconditioned group when specifically designed shoes were worn; no significant changes were observed in the non-deconditioned individuals. The deconditioned group displayed longer step length during the outdoor 30-min ambulation test. In the non-deconditioned group, the use of specific shoes correlated to better performance in terms of gait speed and cadence. These data, although preliminary, seem to support the hypothesis that shoes specifically conceived and designed for counteracting some of the known functional limitations in subjects with obesity allow for a smoother, more stable and possibly less fatiguing gait schema over time.

## 1. Introduction

Footwear conditions influence gait variables. This is of particular importance in subjects experiencing difficulties in deambulation and balance, such as with frail, elderly, and obese subjects. For the latter, finding appropriate footwear that meet comfort and safety expectations is sometimes difficult. Their options rely often on sports footwear capable of accommodating a wider foot, or on orthopaedic shoes for specific foot pain conditions, but choice in-between for everyday life shoes appears limited. Gait in subjects with obesity has been studied with 3D motion capture and it is known that subjects with obesity walk slower than lean individuals, with reduced step length and frequency, wider stance, reduced hip and knee flexion and increased ankle plantarflexion [1,2,3,4]. Joint loading is increased during walking and obesity elevates the risk for musculoskeletal disorders such as osteoarthritis, low back pain, soft tissue injury, tendinitis and plantar fasciitis [5,6]. Musculoskeletal injuries represent a frequent cause of dropping out of physical activity programs. However, despite being a critical source of biomechanical loading, walking remains a highly recommended form of exercise for the management of obesity. Comfortable and adequate shoes represent, together with the prescription of a safe adapted physical activity, an important means to achieve the recommended physical activity target volume.

Literature on shoes specific for obese individuals is limited. Peduzzi de Castro et al. [7] tested the effects of two pressure relief insoles developed for people carrying an extra-weight, like backpackers and subjects with obesity, based on the ground reaction forces and plantar pressure peaks during gait. They demonstrated that insoles showed positive effects for either the plantar pressure distribution or the ground reaction forces parameters. Russel et al. [8] showed by means of gait analysis positive effects of a prophylactic wedged insole for reducing the magnitude of the load on the knee’s medial compartment in obese women who are at risk for knee osteoarthritis development. Griffon et al. [9] demonstrated that the orthosis intervention at foot level significantly improved the ambulatory performances of obese individuals during the 6-min walking test (6MWT), reduced the perception of fatigue and the postural changes occurring after the 6MWT.

Despite the widespread use of 6MWT in clinical studies, the mere output of the test is the distance walked. Additional subject-specific factors such as stride length, velocity or body posture, granularity of overall gait patterns or body segment kinematics, and gait parameters are not measured during the test. In spite of the increasing body of evidence for the use of wearable sensors during gait, their use during the 6MWT (i6MWT: instrumented 6MWT) is not common [10]. i6MWT provides a comprehensive assessment of gait without adding further burden to the patient and represents an alternative to traditional gait analysis and postural control assessment. Those, in fact, require expensive equipment, are time-consuming and provide detailed information only for a very limited number of consecutive gait cycles [11]. Considering the hundreds of steps taken during the 6MWT, the information that can be extracted from this clinical test is potentially very valuable.

Thus, the aim of the present study was to compare the performance of shoes specifically designed for subjects with obesity with everyday sneakers during i6MWT and an outdoor 30-min ambulation in a group of subjects with obesity using an existing single wearable device.

## 2. Materials and Methods

### 2.1. Participants

A sample of 23 obese individuals (OG, 6 males, 17 females, mean age 58.96 years, BMI > 30 kg/m^2^) admitted for a comprehensive multidisciplinary rehabilitation program at the Istituto Auxologico Italiano, Piancavallo (VB, Italy), were recruited for the study on a voluntary basis. At the time of the experimental tests, all of them were free from any acute musculoskeletal, neuromuscular, psychological and/or cardiopulmonary conditions able to significantly affect their walking abilities and postural control.

According to the value of 6MWt distance, participants were classified into two groups: deconditioned (n = 13) and non-deconditioned patients (n = 10). The patient is classified as deconditioned if the 6MWT walked distance is lower than 400 m; in this case patient is considered sarcopenic or potentially frail [12]. If the distance walked during 6MWT is over 400 m, the patient is classified as non-deconditioned. Their anthropometric features are reported in Table 1.

Each participant was evaluated by means of a questionnaire related to the comfort related to each model of shoes and instrumentally during the i6MWT and an outdoor walking test. The tests were repeated in different sessions: in the first session, with his/her daily sneakers and in the second session with shoes specifically designed for people with obesity. The second session was performed the day after the first one, asking the individuals to wear the specifically designed shoes during their normal daily activities.

The questionnaire related to the comfort was a 7 items self-assessment questionnaire:

- Rearfoot pain
- Midfoot pain
- Forefoot pain
- Comfort at rest
- Comfort during walking
- Stability
- Safety

The score of each item is from 0 (absence) and 3 (presence).

All participants were required to sign a written informed consent form, in which the details of the experimental tests were reported. The study was carried out in compliance with the World Medical Association Declaration of Helsinki and its later amendments.

### 2.2. Data Collection and Processing

For the i6MWT and the outdoor 30-min outdoor ambulation test, a single miniaturized inertial sensor (G-Sensor^®^, BTS Bioengineering, Milan, Italy), previously validated for investigations on gait in unaffected individuals and people with several pathological conditions [13,14,15,16,17] was placed on participants’ lower backs, approximately at the L4-L5 vertebrae position. The sensor, which is sized 70 mm × 40 mm × 18 mm and weighs 37 g, was built with a triaxial accelerometer 16 bit/axes with multiple sensitivity (±2, ±4, ±8, ±16 g), a triaxial magnetometer 13 bit (±1200 mT) and a triaxial gyroscope 16 bit/axes with multiple sensitivity (±250, ±500, ±1000, ±2000 °/s).

The i6MWT was performed indoors, along a long, flat, undisturbed 30-m hospital corridor with the length marked every 5 m; turnaround points were marked with a cone. Patients were instructed to walk as fast as they could. Encouragement was given every minute during the test until subject exhaustion using only standardized phrases as specified in the “ATS Statement: Guidelines for the Six-minute Walk Test” [18]. Chest pain, severe dyspnoea, physical exhaustion, muscle cramps, sudden gait instability or other signs of severe distress were additional criteria for stopping the test [18].

After a 15-min recovery, participants performed a supervised outdoor 30-min ambulation test in the hospital park. Participants were asked to refrain from speaking throughout the test.

In each test, acceleration data, acquired at 100 Hz frequency, were transmitted via Bluetooth to a PC and processed using dedicated software (BTS^®^ G-Studio, BTS Bioengineering S.p.A.; ver. 3.2.20) which performs data acquisition, elaboration, reporting and storage. The software used is BTS G-Studio has a specific protocol capable of analysing the i6MWT and gait test, which automatically generates a report for each trial. The first 5 s of acquisition (during which the subject was asked to stand still) were used to verify the orientation of the sensor and then use such information to correct the acceleration vectors data during the acquired trials.

Based on the raw acceleration data, the main spatio-temporal parameters were calculated following the approaches described by literature for each stride [15,19,20] in each session. As for the i6MWT the values of walked distance, gait speed, step length and cadence were computed during the entire test; as for the outdoor 30-min gait test, the values of gait speed, step length and cadence were computed during the early (1st minute) and last (30th minute) segment of the test.

### 2.3. Obesity-Specific Shoes

**Figure 1:**
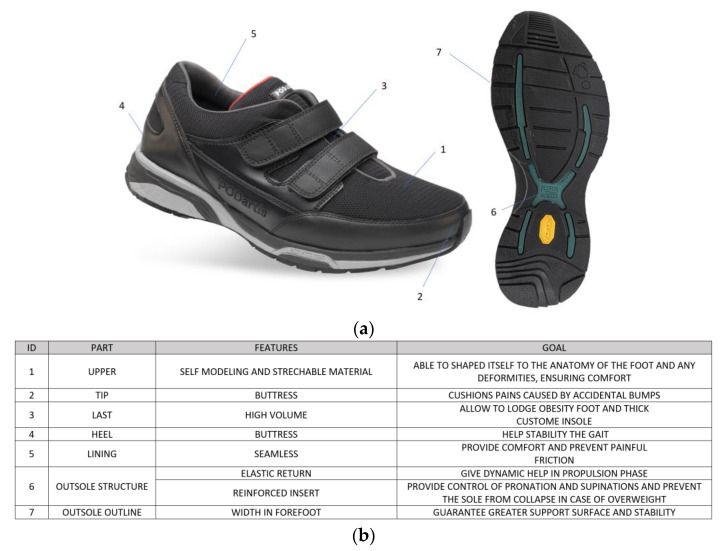
Image related to the obesity-specific shoes (**a**) and description of key elements of obesity-specific shoes with the role of each part (**b**).

The shoes specifically designed for obese individuals (Figure 1) were developed in order to decrease the foot hyper-pronation with valgus of the knees, to compensate the lack of the natural shock absorber given by the arch of the foot (collapsed due to excess weight) and by the heel (the tissues have an excess of fat) and to accommodate the foot extra-volume (usually, subjects with obesity wear 1–2 size larger shoes).

**Figure 2:**
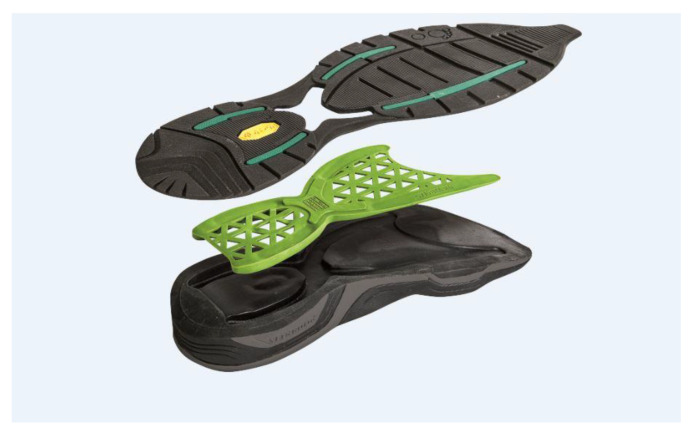
Details of the sole of the obesity-specific shoes used in the study.

Their sole is composed of rubber tread with high resistance to abrasion, an EVA midsole (light material with shock absorber properties able to cushion the impact between foot and ground) and an insert in composite fibers with high resistance and elasticity. In the medial and lateral part of the heel, the sole insert has 2 piers of 16 mm that prevent excessive pronation and supination and at the same time the collapsing of the sole. This elastic insert in composite fibers also has the ability to deform itself during the stance phase, accumulating energy, and to release it during the push off phase. The sole is displayed in detail in Figure 2.

These shoes are able to provide propulsive boost, support and stabilization to the foot.

### 2.4. Statistical Analysis

All the previously defined parameters were computed for each participant in the two sessions and used for analysis.

The Kolmogorov–Smirnov test was necessary to verify if the parameters were normally distributed. Because assumptions of normality were fulfilled, media and standard deviations relating to all indices were calculated for the obese groups (deconditioned and non-deconditioned group) of participants.

A repeated measure ANOVA was performed on the data of i6MWT with the within-subject factor of shoes (daily sneakers vs. specific shoes) and the between-subject factor of group (deconditioned vs. non-deconditioned). A repeated measure ANOVA was performed on the data of the outdoor 30-min ambulation test with the within-subject factor of shoes (daily sneakers vs. specific shoes) and the between-subject factor of time (1st minute vs. 30th minute). Post-hoc tests were performed where appropriate by applying Fisher’s correction for the significance threshold. A significance level of 0.05 was implemented throughout. The statistical analysis was performed using Minitab^®^ (version 18.1, State College, PA, USA).

## 3. Results

Out of the total sample of 23 subjects, 7 were men and 16 were women. The age and anthropometric characteristics of the two study groups are shown in Table 1; their characteristics were not significantly different between groups.

In Table 2 the median and range values of the score for each item of the questionnaire related to comfort for the two groups are reported for the two sessions (daily sneakers vs. specific shoes). It is possible to observe that in the deconditioned group no differences appeared in terms of pain between the two sessions (daily sneakers vs. specific shoes), while as for the other items (comfort stability and safety) the specific shoes seemed to show the higher score. In the non-deconditioned group only, stability and safety appeared to have the higher score in the session when the specific shoes were worn.

In Table 3 the mean and the standard deviations of i6MWT parameters for the entire test of the two groups are reported for the two sessions (daily sneakers vs. specific shoes).

**Figure 3:**
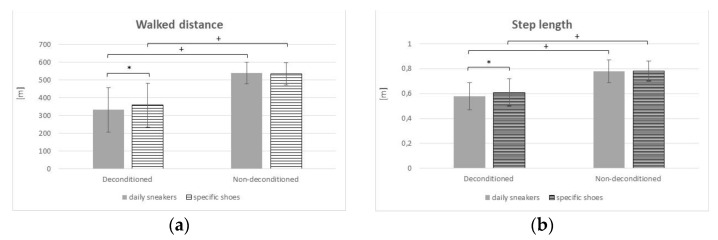
Mean and standard deviation of walked distance (**a**) and step length (**b**) during the i6MWT of the two groups are reported for the two sessions (daily sneakers vs. specific shoes). * = *p* < 0.05, statistically significant in the post hoc comparison daily sneakers vs. specific-obesity shoes; + = *p* < 0.05, statistically significant in the post hoc comparison deconditioned vs. non-deconditioned group.

*Walked distance* (Figure 3a): The main effect of Group [F(1, 38) = 38.84; *p* < 0.001; partial η^2^ = 0.5054] was significant, with lower value for the deconditioned patients and also the main effect of Shoes was significant [F(1, 38) = 0.11; *p* = 0.039; partial η^2^ = 0.1030], with lower value with the personal shoes; the Group × Shoes interaction was confirmed as significant [F(1, 38) = 1.19; *p* = 0.032; partial η^2^ = 0.1070].

*Gait speed*: The main effect of Group [F(1, 38) = 46.60; *p* < 0.001; partial η^2^ = 0.5508] was significant, with lower value for the deconditioned patients, while the main effect of Shoes was not significant [F(1, 38) = 0.05; *p* = 0.821; partial η^2^ = 0.0014]; the Group × Shoes interaction revealed to be not significant [F(1, 38) = 0.09; *p* = 0.770; partial η^2^ = 0.0023].

*Step length* (Figure 3b): The main effect of Group [F(1, 38) = 39.66; *p* < 0.001; partial η^2^ = 0.5107] was significant, with lower value for the deconditioned patients and also the main effect of Shoes was significant [F(1, 38) = 0.03; *p* = 0.043; partial η^2^ = 0.1034], with lower value with the personal shoes; the Group × Shoes interaction was confirmed as significant [F(1, 38) = 1.09; *p* = 0.041; partial η^2^ = 0.1008].

*Cadence*: The main effect of Group [F(1, 38) = 8.20; *p* = 0.007; partial η^2^ = 0.1775] was significant, with lower value for the deconditioned patients, while the main effect of Shoes was not significant [F(1, 38) = 0.44; *p* = 0.513; partial η^2^ = 0.0114]; the Group × Shoes interaction revealed to be not significant [F(1, 38) = 1.08; *p* = 0.306; partial η^2^ = 0.0276].

Values of parameter in the 1st and 6th segment of 6MWT, where no statistical results were obtained, are not reported.

In Table 4 and Table 5 the mean and the standard deviations of the parameters during the outdoor 30-min gait test were reported during the early (1st minute) and last (30th minute) segment of the test during the two sessions (daily sneakers vs. specific shoes) for the deconditioned (Table 3) and for the non-deconditioned group (Table 4).

Here, the results for the deconditioned group are reported (Table 4):

*Gait speed*: The main effects of shoes [F(1, 48) = 0.07; *p* = 0.798; partial η^2^ = 0.0014] was not significant, the main effect of Time [F(1, 48) = 6.15; *p*= 0.017; partial η^2^ = 0.1135] was significant; the Time × Shoes interaction revealed to be not significant [F(1, 48) = 1.06; *p* = 0.309; partial η^2^ = 0.0216]

**Figure 4:**
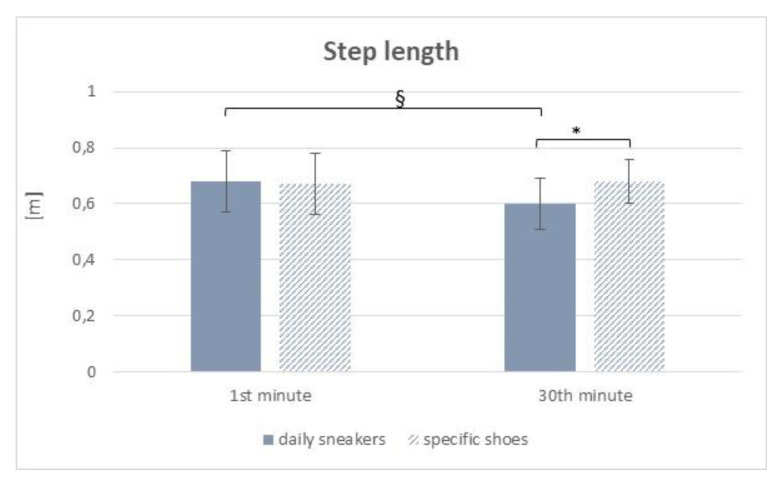
Mean and standard deviation of step length during the outdoor 30-min gait test for the deconditioned group during the early (1st minute) and last (30th minute) segment of the test during the two sessions (daily sneakers vs. specific shoes). * = *p* < 0.05, statistically significant in the post hoc comparison daily sneakers vs. specific shoes; § = *p* < 0.05, statistically significant in the post hoc comparison 1st minute vs. 30th minute.

*Step length* (Figure 4): The main effect of shoes [F(1, 48) = 0.27; *p* = 0.043; partial η^2^ = 0.1056] was significant, with lower value with the daily sneakers, the main effect of Time was significant [F(1, 48) = 0.15; *p* = 0.046; partial η^2^ = 0.1032], with lower values as for the 30th minute; the Group × Shoes interaction was confirmed as significant [F(1, 48) = 0.39; *p* = 0.041; partial η^2^ = 0.1081], with significant difference in the comparison between the 1st and the 30th minute with the daily sneaker and between the daily sneaker and the specific-obesity shoes at the 30th minute.

*Cadence*: The main effects of shoes [F(1, 48) = 0.02; *p* = 0.888; partial η^2^ = 0.0004] and Time [F(1, 48) = 2.84; *p* = 0.099; partial η^2^ = 0.0558] were not significant; the Time × Shoes [F(1, 48) = 0.05; *p* = 0.819; partial η^2^ = 0.0011] interaction was not significant.

Below, the results for the non-deconditioned group are reported (Table 5):

*Gait speed*: The main effect of shoes [F(1, 36) = 0.10; *p* = 0.043; partial η^2^ = 0.1028] was significant, with lower values with daily sneakers, the main effect of Time [F(1, 36) = 1.85; *p* = 0.182; partial η^2^ = 0.0489] was not significant and the Time × Shoes [F(1, 36) = 3.21; *p* = 0.082; partial η^2^ = 0.0819] interaction was not significant.

*Step length*: The main effects of shoes [F(1, 36) = 0.02; *p* = 0.902; partial η^2^ = 0.0004] and Time [F(1, 36) = 0.01; *p* = 0.910; partial η^2^ = 0.0004] were not significant; the Time × Shoes interaction was confirmed to be not significant [F(1, 36) = 0.001; *p* = 0.972; partial η^2^ = 0.0001].

*Cadence*: The main effects of shoes [F(1, 36) = 0.15; *p* = 0.046; partial η^2^ = 0.1060] was significant, with lower values with daily sneakers, the main effect of Time [F(1, 36) = 1.89; *p* = 0.178; partial η^2^ = 0.0498] was not significant and the Time × Shoes [F(1, 36) = 4.27; *p* = 0.705; partial η^2^ = 0.0040] interaction was not significant.

## 4. Discussion

The influence of obesity on gait biomechanics has been extensively investigated with quantitative gait analysis, but far less attention has been devoted to the effects of footwear on walking capacity in individuals with obesity.

The purpose of this study was to compare the comfort and the performance of shoes specifically designed for subjects with obesity with everyday sneakers during i6MWT and outdoor 30-min ambulation test in a group of obese subjects using a single wearable device. We wanted to test whether subjects with obesity could improve distance walked during the 6MWT and reduce fatigue using shoes specifically designed for them as compared to habitual shoes. Whereas the technology of new shoes for subjects with obesity remains the focus of this paper, its originality relates to the simple, indirect method used to assess their performance by means of commonly used clinical and ecological tests instrumented with a single wearable device.

Our results showed that in terms of comfort the specifically designed shoes that were worn displayed the higher score, in particular in the deconditioned group; no differences appeared in terms of pain reduction.

As for the instrumental tests, during the i6MWT, the distance walked and step length significantly increased in the deconditioned group when specifically designed shoes were worn. No differences were found in terms of gait speed and cadence, even if a trend towards an increase could be observed. No significant changes were observed in the non-deconditioned individuals. As for the results related to the outdoor 30-min ambulation test, the deconditioned group displayed better performance when using specific shoes, with longer step length as compared to that obtained using daily sneakers. While no statistically significant differences were found in the comparison between the beginning (1st minute) and the end (30th minute) of the outdoor ambulation test when using specific shoes, a significant reduced step length was observed at the 30th minute of walking outdoor when using daily sneakers. In the non-deconditioned group, the use of specific shoes correlated to better performance in terms of gait speed and cadence, with no differences in terms of time (1st vs. 30th minute). Those data seem to support the hypothesis that shoes specifically conceived and designed for counteracting some of the known functional limitations in subjects with obesity allow a smoother, more stable and possibly less fatiguing gait schema over time.

These preliminary results show encouraging data about the use of the specific shoes tested as compared to daily sneakers in a group of individuals with obesity. In particular, the positive effects are evident in the deconditioned group, characterised by more limited parameters as compared to the non-deconditioned group.

Our study presents with some limitations. Firstly, the small number of participants for each group (deconditioned and non-deconditioned) with a wide age range (from 35 to 83 years), which result in limited strength of the statistical findings. A larger and more homogeneous group of patients in terms of age could strengthen the results. Secondly, both males and females were recruited for this study in order to improve the generalizability of the results. Combining male and female subjects, though, introduces a source of potential variability, as obesity modifies the body geometry by adding mass to different regions and dissimilar fat distribution in males and females could produce gender-related effects. However, with our sample it was not possible to consider them separately. Thus, potential differences between males and females who are obese will need further study. Another limitation is related to the absence of a stratification of the participants in terms of the severity of obesity. Future larger studies for each obesity class are needed for a deeper understanding of the effects of specific shoes, as effects may be related to the degree of severity of obesity.

## Figures and Tables

**Table 1:** Participant characteristics.

	Deconditioned	Non-Deconditioned	*p*-Value
	(n = 13)	(n = 10)	
Gender, n (%)			
Male	3 (23.1%)	4 (40%)	
Age (years)	63.78 ± 8.67	58.55 ± 8.61	0.989
Height (m)	1.59 ± 0.07	1.64 ± 0.07	0.092
Body mass Index (kg/m^2^)	41.34 ± 3.69	39.82 ± 3.17	0.118

**Table 2:** Median (minimum and maximum) values of the score for the questionnaire related to comfort for the two groups are reported for the two sessions (daily sneakers vs. specific shoes). * = *p* < 0.05, statistically significant in the post hoc comparison daily sneakers vs. specific-obesity shoes.

	Deconditioned		Non-Deconditioned	
	Daily Sneakers	Specific Shoes	Daily Sneakers	Specific Shoes
Rearfoot pain	0 (0–3)	0 (0–2)	0 (0–1)	0 (0–1)
Midfoot pain	0 (0–3)	0 (0–3)	0 (0–1)	0 (0–1)
Forefoot pain	0 (0–3)	0 (0–1)	0 (0–1)	0 (0–1)
Comfort at rest	2 (1–3) *	3 (2–3)	2 (0–3)	3 (2–3)
Comfort during walking	2.5 (0–3) *	3 (2–3)	3 (0–3)	3 (2–3)
Stability	2 (0–3) *	3 (2–3)	2 (0–3)	3 (3–3) *
Safety	2 (0–3) *	3 (2–3)	2 (0–3)	3 (2–3) *

**Table 3:** Mean and standard deviation of i6MWT parameters of the two groups are reported for the two sessions (daily sneakers vs. specific shoes). * = *p* < 0.05, statistically significant in the post hoc comparison daily sneakers vs. specific-obesity shoes; + = *p* < 0.05, statistically significant in the post hoc comparison deconditioned vs. non-deconditioned group.

	Deconditioned		Non-Deconditioned	
	Daily Sneakers	Specific Shoes	Daily Sneakers	Specific Shoes
Walked distance (m)	332.15 (125.82) +	358.32 (122.04) *+	539.14 (59.73)	533.53 (62.36)
Gait speed (m/s)	1.08 (0.28) +	1.12 (0.28) +	1.62 (0.18)	1.61 (0.19)
Step length (m)	0.58 (0.11) +	0.61 (0.11) *+	0.78 (0.09)	0.78 (0.08)
Cadence (step/min)	109.69 (17.53) +	115.91 (12.82) +	124.03 (5.69)	122.64 (6.69)

**Table 4:** Mean and standard deviation of parameters during the outdoor 30-min gait test for the deconditioned group during the early (1st minute) and last (30th minute) segment of the test during the two sessions (daily sneakers vs. specific shoes). * = *p* < 0.05, statistically significant in the post hoc comparison daily sneakers vs. specific shoes; § = *p* < 0.05, statistically significant in the post hoc comparison 1st minute vs. 30th minute.

Deconditioned				
	Daily Sneakers		Specific Shoes	
	1st Minute	30th Minute	1st Minute	30th Minute
Gait speed (m/s)	0.92 (0.28)	0.90 (0.18)	0.96 (0.28)	0.99 (0.32)
Step length (m)	0.68 (0.61)	0.60 (0.08) §*	0.67 (0.08)	0.68 (0.07)
Cadence (step/min)	87.04 (31.10)	90.96 (14.09)	89.40 (26.91)	93.52 (30.75)

**Table 5:** Mean and standard deviation of parameters during the outdoor 30-min gait test for the non-deconditioned group during the early (1st minute) and last (30th minute) segment of the test during the two sessions (daily sneakers vs. specific-obesity shoes). * = *p* < 0.05, statistically significant in the post hoc comparison daily sneakers vs. specific-obesity shoes.

Non-Deconditioned				
	Daily Sneakers		Specific Shoes	
	1st Minute	30th Minute	1st Minute	30th Minute
Gait speed (m/s)	1.13 (0.36) *	1.14 (0.22) *	1.28 (0.28)	1.23 (0.32)
Step length (m)	0.75 (0.10)	0.76 (0.10)	0.76 (0.25)	0.76 (0.25)
Cadence (step/min)	92.63 (27.38) *	95.85 (16.78) *	104.98 (24.76)	99.91 (19.91)

## Data Availability

Data available on request due to restrictions e.g., privacy or ethical.
